# Interleukin-13 suppresses interleukin-10 via inhibiting A20 in peripheral B cells of patients with food allergy

**DOI:** 10.18632/oncotarget.13107

**Published:** 2016-11-04

**Authors:** Ming-yang Li, Min Zhu, En-qiang Linghu, Fan Feng, Bing Zhu, Cheng Wu, Ming-zhou Guo

**Affiliations:** ^1^ Department of Gastroenterology and Hepatopathy, Chinese PLA General Hospital, Beijing, 100853, China; ^2^ Department of Medical Oncology, Division of South Building, Chinese PLA General Hospital, Beijing, 100853, China; ^3^ Department of Pharmacy, General Hospital of Shenyang Military Command, Shenyang 110016, PR China; ^4^ Liver Failure Treatment and Research Center, 302nd Military Hospital, Beijing 100039, China; ^5^ Department of Digestive Endoscopy, Division of South Building, Chinese People's Liberation Army General Hospital, Beijing 100853, China

**Keywords:** interleukin-13, histone deacetylase, immunity, immune regulation

## Abstract

The regulatory B cells (Breg) are important in the body immunity. The differentiation process of Breg is not fully understood yet. Ubiquitin A20 has immune regulatory functions. This study aims to investigate the role of A20 in the regulation of interleukin (IL)-10 in B cells. In this study, B cells were isolated from the peripheral blood samples of healthy subjects and patients with food allergy (FA). The B cells were analyzed by flow cytometry, real time RT-PCR, Western blotting and chromatin immunoprecipitation. We observed that the frequency of Breg and the levels of A20 in B cells were markedly lower in FA patients than in healthy controls. *In vitro* deletion of A20 compromised the expression of IL-10. B cells in FA patients showed higher levels of histone deacetylase (HDAC)-11 than in healthy subjects. Exposure to IL-13 in the culture induced high levels of HDAC11 in B cells. IL-13 also repressed the expression of A20 in B cells, in which HDAC11 played a critical role via inducing the chromatin remoldeling at the IL-10 promoter locus. Mice with A20-deficient B cells are prone to FA. In summary, ubiquitin A20 can increase the IL-10 expression in B cells, which can be affected by the IL-13-induced HDAC11. To inhibit HDAC11 may have therapeutic potential for FA.

## INTRODUCTION

B cells (BC) are a major fraction of the immune cells in the body. Apart from producing antibodies, B cells also have an immune regulatory function via producing immune regulatory cytokines, such as interleukin (IL)-10 [1] or transforming growth factor (TGF)-β [2, 3]. IL-10 can be produced by several cell types, including dendritic cells, T cells, BCs and monocytes, etc [4]. It is reported that the IL-10^+^ BCs had immune regulatory function and the frequency of IL-10^+^ BC was less in several immune disorders, including allergic diseases [5, 6], which can be restored by specific allergen immunotherapy [7]. The regulation of IL-10 in B cells has not been fully understood.

Food allergy (FA) is an abnormal immune response that is induced by the immune cells in the intestine over reacting to the innocent food allergens [8]. FA is mainly mediated by immunoglobulin (Ig)E. IgE binds to the high affinity receptor of mast cells to make the mast cells sensitized. Re-exposure to specific antigens triggers the sensitized mast cells to release allergic mediators to induce allergic response [8]. In general, immune response is tightly regulated by the immune system, mainly regulated by regulatory T cells or/and regulatory B cells [9]. Because of unknown mechanism, the frequency or/and functions of the immune regulatory cells are reduced or compromised [10]; the underlying mechanism remains to be further investigated.

The ubiquitin E3 ligase A20 (A20, in short) is also called tumor necrosis factor (TNF)-α-induced protein 3. This protein in humans is encoded by the *TNFAIP3* gene [11]. A20 is a zinc finger protein, and has been shown to inhibit immune inflammation via suppressing the NF-kappa B activation and the TNF-mediated apoptosis [12]. It is suggested that A20 has an immune regulatory function [13]. Most body cells express A20, which can be up regulated by lipopolysaccharide (LPS). One of the functions of A20 is its protection from allergic disorders. The dysregulation of A20 may lead to allergic disorders [14].

The histone deacetylases (HDAC) are an enzyme family playing a critical role in the regulation of gene transcription of a large number of molecules. HDACs are able to catalyze the hydrolysis of acetyl groups on lysine residues of histones, causing the condensation and coiling of chromosomal DNA around histones, and therefore regulating gene expression. It is reported that inhibitors of HDACs are beneficial to patients with allergic disorders [15], indicating that HDACs are associated with the pathogenesis of allergy. Recent studies also revealed that HDAC11 inhibited the expression of IL-10 and affected cells' tolerogenic property [16].

One of the pathological features of allergic diseases is the over-production of T helper (Th) 2 cytokines, including IL-4, IL-5, IL-13, etc., in the sera and the local tissue [17]. Apart from being involved in the allergic responses, IL-13 is also involved in regulating a large number of gene transcription [18]. Recent reports revealed that IL-13 repressed the gene transcription of thrombospondin-1 in B cells to compromise BC's tolerogenic property [19]. Based on the above information, we hypothesize that the Th2 cytokine IL-13 may interfere with the IL-10 expression in B cells via regulating the levels of HDAC11 and A20. Thus, we carried out this study. The results indicate that the A20 levels and IL-10 levels in peripheral B cells were lower in food allergy (FA) patients than in healthy subjects. Exposure of B cells to IL-13 *in vitro* increased the expression of HDAC11, which repressed the expression of A20 and IL-10 in the B cells. HDAC11 may be a potential therapeutic target for food allergy.

## RESULTS

### The levels of A20 are lower in IL-10^+^ B cells of FA patients

**Figure 1:**
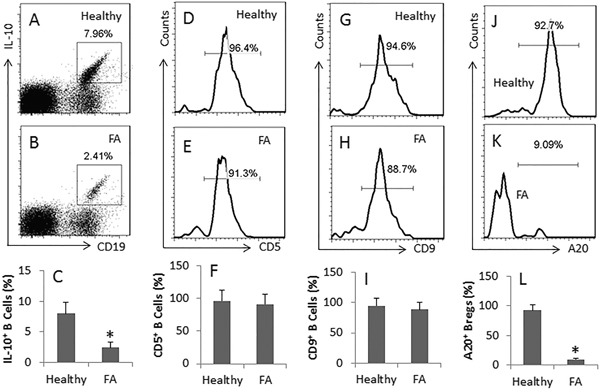
Assessment of A20 expression in peripheral Bregs **A-B.**, the dot plots show the frequency of CD19^+^ IL-10^+^ Bregs in peripheral blood mononuclear cells collected from 20 healthy subjects (Healthy) (A) and 20 FA patients (FA) (B). The flow cytometry histograms show the frequency of CD5^+^ cells **D, E.**, CD9^+^ cells **G, H.** and A20^+^ cells **J, K.** in the gated cells of A and B, respectively. The bar graphs (mean ± SD) show the summarized data of A and B **C.,** D and E **F.,** G and H **I.** and J and K **L.** *, p<0.01, compared with the healthy group. The presented flow cytometry data are from one experiment out of 20 independent experiments.

It is suggested that dysregulation of regulatory T cells (Treg) or/and regulatory B cells (Breg) plays an important role in the pathogenesis of intestinal allergy [3]. While the association between Breg dysregulation and the pathogenesis of allergy is less clear. We wondered if the population of Bregs was deregulated in food allergy (FA) patients. To this end, we collected peripheral blood samples from FA patients and healthy subjects, and analyzed by flow cytometry. The results showed that the frequency of IL-10^+^ Bregs was significantly less in FA patients than in healthy subjects. The IL-10^+^ Bregs were CD5 and CD9 positive as well; but no significant difference was detected on CD5^+^ and CD9^+^ cells between healthy and FA patients. (Figure 1A–1I). We further analyzed the frequency of A20^+^ Bregs by flow cytometry. The results showed much less A20^+^ Bregs were detected in FA patients as compared with healthy subjects (Figure 1J-1L).

### A20 is required for IL-10 expression in B cells

**Figure 2:**
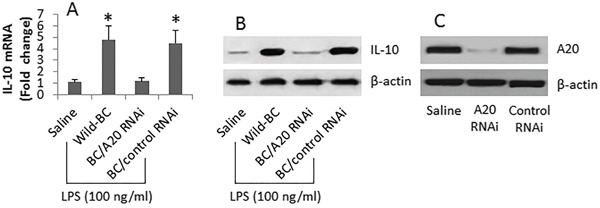
A20 is associated with IL-10 expression in B cells B cells were isolated from 6 healthy subjects and cultured for 3 days in the presence of LPS (100 ng/ml) and anti-CD40 (10 ng/ml). **A-B.**, the bars indicate the IL-10 mRNA levels, the immune blots indicate the IL-10 protein levels, in the B cells after the treatment denoted on the X axis. **C.**, the Western blots show the A20 gene knockdown results in the B cells. The data of bars are presented as mean ± SD. *, p<0.01, compared to the saline group. The data are representatives of 3 independent experiments.

To elucidate if the dysregulation of A20 in B cell is associated with the reduced frequency of Bregs in FA patients, we treated B cells (from healthy individuals) with LPS in the culture. The results showed that LPS markedly increased the expression of IL-10 in B cells (Figure 2A-2B), which was inhibited by knocking down the A20 in B cells (Figure 2C). The results suggest that A20 plays an important role in the expression of Il-10 in B cells.

### HDAC11 is involved in the IL-13-induced suppression of A20 in B cells

**Figure 3:**
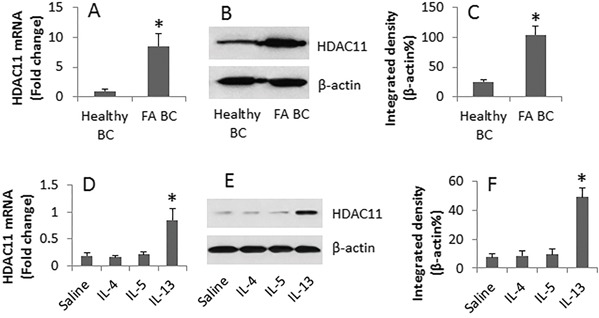
IL-13 increases HDAC11 in B cells B cells were obtained from 6 healthy subjects and 6 FA patients. **A-C.**, the cell extracts were prepared immediately after isolation. **D-F.**, a portion of healthy B cells were cultured in the presence of saline or IL-4 (100 ng/ml), or IL-5 (100 ng/ml), or IL-13 (100 ng/ml) and anti-CD40 (10 ng/ml) for 48 h. The cell extracts were analyzed by RT-qPCR and Western blotting. A and D, the bars indicate the mRNA levels of HDAC11. B and E, the immune blots indicate the protein levels of HDAC11. C and F, the bars indicate the integrated density of the blots in B and E. Samples from individual subjects were analyzed separately. *, p<0.01, compared to the Healthy B cells (A and C), or the saline group (D and F).

Published data indicate that HDAC11 suppresses IL-10 expression [16]. From the data reported above, we inferred that HDAC11 might be involved in the IL-10 suppression in FA B cells. To test the inference, we analyzed the levels of HDAC11 in FA B cells and B cells from healthy individuals. The results showed that the HDAC11 was higher in FA B cells than in B cells from healthy individuals (Figure 3A-3C). Prompted by published data that levels of IL-13 are higher in allergic diseases and IL-13 modulates other gene transcription [18], we exposed B cells from healthy individuals to Th2 cytokines IL-4, or IL-5, or IL-13 in the culture. Indeed, the levels of HDAC11 were markedly increased in the B cells after exposing to IL-13, but not to IL-4 or IL-5 (Figure 3D-3F).

**Figure 4:**
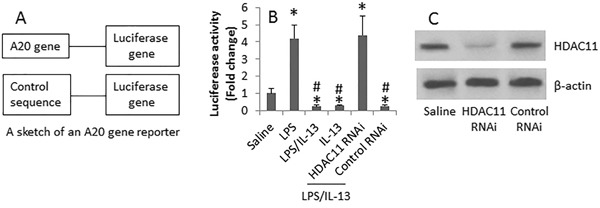
HDAC11 is involved in the inhibition of A20 gene activity by IL-13 in B cells B cells were isolated from 18 healthy subjects. The cells were transfected with an A20 gene reporter **A.** and cultured as denoted on the X axis of panel **B.** in the presence of anti-CD40 (10 ng/ml) for 16 h. The bars (mean ± SD) of panel B indicate the luciferase activity in the B cells. LPS (100 ng/ml). IL-13 = 100 ng/ml. **C.**, the immune blots show the HDAC11 RNAi results. *, p<0.01, compared to the saline group. #, p<0.01, compared with the LPS group.

Based on the data of Figure 2 indicate that A20 is required in the expression of IL-10 in B cells, we reasoned that HDAC11 might mediate the suppression of A20 expression in B cells. To test this, we transfected an A20 reporter gene into B cells. The cells were exposed to LPS or/and IL-13 for 16 h. As analyzed by luciferase assay, exposure to LPS significantly increased the A20 gene activity, which was suppressed in the cells exposed to both LPS and IL-13. Knocking down the gene of HDAC11 abolished the suppressive effects of LPS/IL-13 on luciferase activity (Figure 4). The data implicate that IL-13 increases HDAC11; the latter mediates the effects of IL-13 on suppression of A20 in B cells.

### HDAC11 mediates the IL-13 repressed IL-10 gene transcription in B cells via inducing chromatin remoldeling at the IL-10 promoter locus

**Figure 5:**
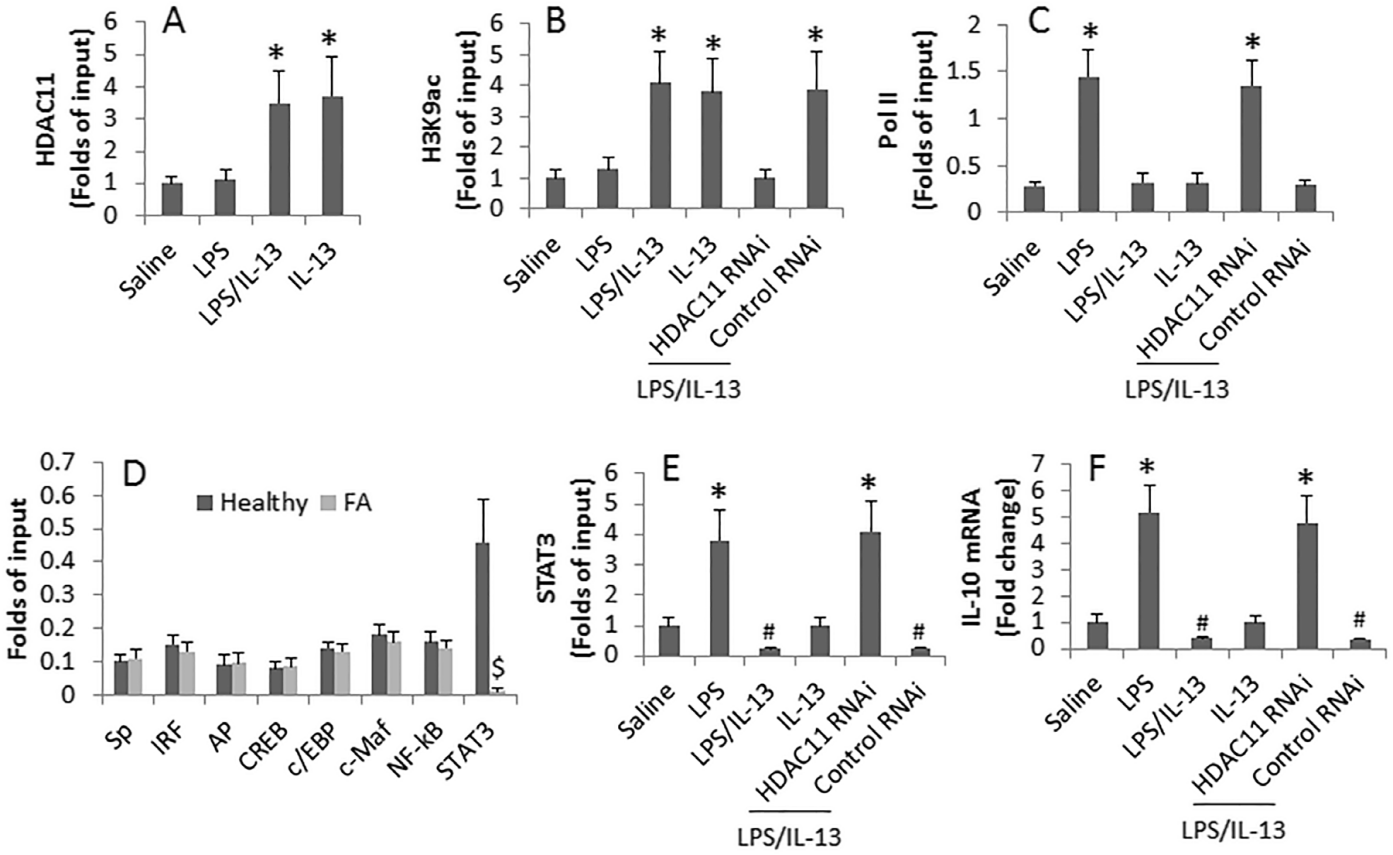
IL-13 induces chromatin remolding at the IL-10 promoter locus in B cells **A-E.**, the levels of HDAC11 (A), acetylated H3K9 (B), Pol II (C), related transcription factors of IL-10 (D) and STAT3 (E) at the IL-10 promoter locus. **F.**, IL-10 mRNA levels in B cells. The peripheral B cells were obtained from 18 healthy subjects and 6 FA patients. B cells were treated in the culture as denoted on the X axis for 16 h (A-C, E-F). LPS (100 ng/ml). IL-13 (100 ng/ml). *, p<0.01, compared to the saline group. #, p<0.01, compared to the LPS alone group. $, p<0.01, compared to the healthy group.

To test the role of HDAC11 in mediating the IL-13-suppressed IL-10 expression in B cells, we isolated B cells from healthy subjects. The cells were cultured for 16 h in the presence of LPS or/and IL-13. As shown by ChIP assay, exposure to IL-13 significantly enriched the levels of HDAC11, acetylated H3K9 and polymerase II at the IL-10 promoter locus (Figure 5A-5C).

Since several transcription factors are related to the IL-10 gene transcription [20], we screened 8 transcription factors in peripheral B cells collected from healthy subjects and FA patients. The results showed that the levels of STAT3 was higher at the IL-10 promoter locus in healthy subjects than that in FA patients, while the levels of the rest 7 transcription factors at the IL-10 promoter locus were not significantly different between healthy group and FA group (Figure 5D). The results implicate that STAT3 is involved in the gene transcription of IL-10 in B cells. We then exposed B cells to IL-13 or/and LPS in the culture. The results showed that exposure to LPS increased the STAT3 levels at the IL-10 promoter locus, which was abolished by the presence of IL-13; the effects of IL-13 were antagonized by knocking down the HDAC11 gene (Figure 5E). The data of Figure 5E were supported by the assessment of IL-10 mRNA in the B cells, which was in parallel to the changes of STAT3 levels at the IL-10 promoter locus (Figure 5F).

### Mice with A20^−/−^ B cells are prone to food allergy

**Figure 6:**
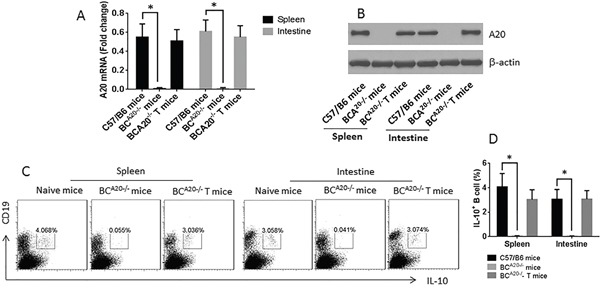
Assessment of IL-10 expression in B cells in the spleen and intestine of wild type mice and BCA20−/− mice **A-B.**, A20 mRNA levels (A) and protein levels (B) in B cells isolated from C57/B6 mice, BC^A20−/−^ mice and BC^A20−/−^ T mice (received A20-sufficient B cell transfer). **C.**, the gated dot plots show the frequency of IL-10^+^ B cells in the spleen and intestine of wild type mice and BC^A20−/−^ mice. **D.**, summarized data of C. Each group consists of 6 mice.

**Figure 7:**
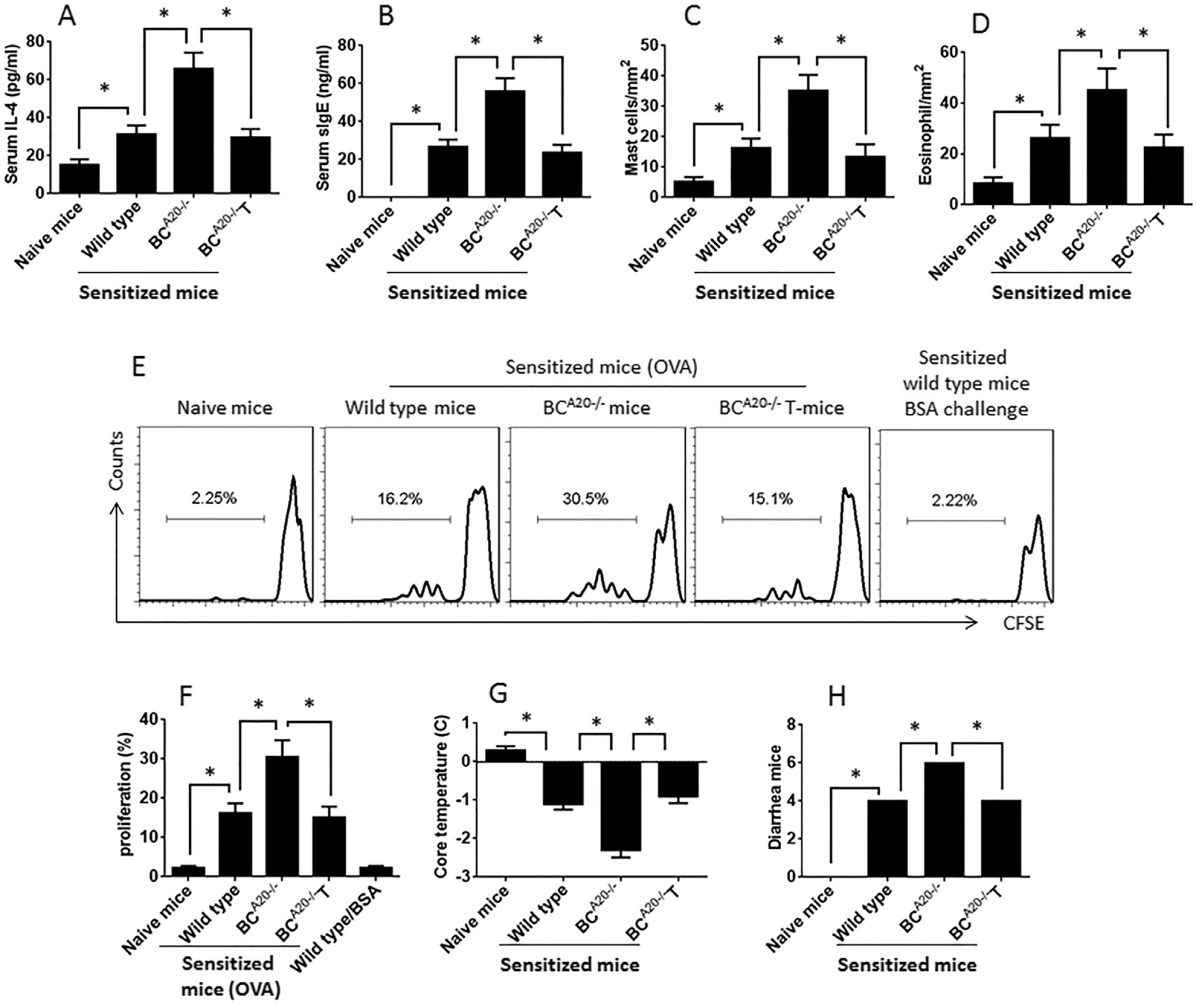
Mice with A20-deficient B cells show higher allergic responses **A-D.**, the bars show the serum levels of IL-4 (A) and specific IgE, cell counts of mast cells (C) and eosinophils (D) in the intestinal mucosa. **E.**, the gated histograms show the CD4^+^ T cell proliferation after culturing with specific antigen (OVA) or BSA in the presence of DC for 3 days. **F.**, the summarized data of E. **G.**, the records of core temperature measured at 30 min after antigen challenge. **H.**, the number of diarrhea mice during 2 h after antigen challenge. The data are presented as mean ± SD. *, p<0.01. Each group consists of 6 mice. Samples from individual mice were processed separately.

To further test the role of A20 in the immune tolerant feature of B cells, we obtained BC^A20−/−^ mice from the Huada Gene Biotech. The BC^A20−/−^ mice showed much less IL-10 expression in B cells in the spleen and intestine (Figure 6A-6B) and less IL-10^+^ B cells in the spleen and intestine (Figure 6C-6D). We sensitized wild type mice and BC^A20−/−^ mice with ovalbumin. The results showed that after sensitization, the wild type mice showed allergic response and inflammation in the intestine, including increases in serum IL-4 and specific IgE, infiltration of eosinophils and mast cells in the intestinal mucosa, antigen specific CD4^+^ T cells in the intestinal mucosa, drop of the core temperature and diarrhea. In BC^A20−/−^ mice, however, the allergic responses were much more severe than that in wild type mice. Reconstituted with wild B cells mitigated the allergic responses in BC^A20−/−^ mice (Figure 7).

## DISCUSSION

It is accepted that the immune tolerance plays a critical role in the maintenance of homeostasis in the intestine [21]; the breakdown of immune tolerance plays a role in the initiation of food allergy [22]. Yet, the pathogenesis of the immune tolerance breakdown is unclear. The present data demonstrate that the dysregulation of the expression of A20 contributes to the dysregulation of IL-10 in B cells of FA patients. The data showed that the levels of the A20 and IL-10 were lower in FA patients than in healthy subjects. Exposure to IL-13, one of the main Th2 cytokines, markedly suppressed the expression of IL-10 in B cells via up regulating HDAC11. Blocking HDAC11 efficiently abrogated the effect of IL-13 on suppressing IL-10 expression in B cells. Mice with knockdown of A20 in B cells showed more severe allergic response. The data demonstrate that the IL-13-inhibited A20 expression in peripheral B cells results in the suppression of IL-10 expression. Since IL-10 is an important molecule in immune tolerance [1], the data suggest that A20 plays an important role in the maintenance of immune tolerance.

IL-10 is one of the immune regulation molecules. Cells expressing IL-10 have immune regulatory functions. The present study found that the frequency of IL-10^+^ BC was lower in FA patients than in healthy subjects. In line with reports of other investigators [23, 24], our results showed that over 90% IL-10^+^ B cells were also CD5 positive and CD9 positive. Since the CD9 molecules are suggested suppressing the skewed T helper cell responses, our data support the notion that the IL-10^+^ CD5^+^ CD9^+^ B cells have immune regulatory properties [23, 24].

The present data also show that the levels of A20 are lower in FA patient B cells than in B cells from healthy individuals. A20 is a molecule having multiple functions. One of which is that A20 plays an important role in the maintenance of the homeostasis in the body. Wang et al reported that ubiquitin-editing enzyme A20 promoted tolerance to lipopolysaccharide in enterocytes [25]. Li et al found that immune responsive gene-1 promoted endotoxin tolerance by increasing A20 expression in macrophages through reactive oxygen species [26]. The deficiency of A20 has been observed in a number of immune disorders. Schuijs et al reported that intestinal epithelial cells expressed A20. The deficiency of A20 promoted intestinal allergy [14]. Li et al observed that exposure to cholera toxin suppressed the expression of A20, which resulted in compromising epithelial barrier function [27]. Vereecke et al found that deficiency of A20 promoted the development of colitis in mice [28]. Thus, we may suggest that the dysregulation of A20 in B cells also contributes to the pathogenesis of FA.

Recent reports indicated that HDAC11 suppressed the expression of IL-10 so as to compromise the immune tolerance [16]. Since our data also showed such a phenomenon that both IL-10 and HDAC11 were deregulated, and A20 was also deregulated, in B cells of FA patients, a connection may exist among these molecules. The inference is supported by the data that the HDAC11 was capable of repressing the expression of A20 in B cells.

Because of that HDAC11 can inhibit the IL-10 expression [16] and IL-10 is an important immune regulatory molecule [29] in the body, to find the causative factors in the up regulation of HDAC11 is of significance. The present results showed high serum levels of IL-13 and high levels of HDAC11 in B cells of FA patients, which suggest a possibility that IL-13 may regulate the expression of HDAC11 in B cells. The inference was supported by the present data, that exposure to IL-13 in the culture markedly up regulated the expression of HDAC11 in B cells. Ooi et al also found that IL-13 is capable of regulating a large number of molecules by regulating the gene transcription [18]. The present data are in line with Ooi et al's reports by showing that IL-13 induced the DNA remolding at the IL-10 promoter locus, which resulted in the repression of IL-10 in B cells.

Considering a shortcoming of the study that the data were only obtained from *in vitro* study with human cells, we developed a mouse food allergy model with BC^A20−/−^ mice and wild type mice. The results supported the data obtained from experiments with human cells and showed that A20 played a critical role in the IL-10 expression in B cells. Short of the IL-10 expression B cells markedly enhanced the allergic response in mice. The results indicate that the A20-sufficient B cells are required in the maintenance of the homeostasis in the body. Similar data were also reported by others. Nagamachi et al als found that A20 deficiency resulted in system inflammation [30]. Hovelmeyer et al observed that A20 deficiency resulted in an excessive production of self-reactive autoantibodies that may contribute to the development of autoimmune disorders [31].

In summary, the present data demonstrate that the levels of A20 and IL-10 are lower, HDAC11 is higher, in FA patients than in healthy subjects. Exposure to IL-13 in the culture increases the expression of HDAC11 in B cells, the latter inhibits the expression of A20 and IL-10 in B cells, which is abolished by blocking the expression of HDAC11. HDAC11 may be a potential therapeutic target for food allergy.

## MATERIALS AND METHODS

### Reagents

The fluorochrome-labeled antibodies for flow cytometry were purchased from eBioscience (San Diego, CA). The antibodies of IL-10, A20, HDAC11, Sp, IRF, AP, CREB, c/EBP, c-Maf, NF-κB, STAT3, HDAC11 shRNA kit and A20 shRNA kit were purchased from Santa Cruz Biotech (Santa Cruz, CA). LPS, ChIP kit and luciferase kit were purchased from Sigma Aldrich (St. Louis., MO). The recombinant IL-4, IL-5, IL-13 and the ELISA kit of IL-4 were purchased from R&D Systems (Minneapolis, MN). The OVA-specific IgE ELISA kit was purchased from the Biomart (Beijing, China). The reagent kits for magnetic cell sorting were purchased from Miltenyi Biotech (San Diego, CA). Reagents for RT-qPCR and Western blotting were purchased from Invitrogen (Carlsbad, CA).

### Ethic statement

The using human tissue and mice in the present study was approved by the Human Ethic Committee and Animal Care Committee at the Chinese PLA General Hospital. All the methods involving humans were carried out in “accordance” with the approved guidelines. A written, informed consent was obtained from each subject.

### Human subjects

In total 32 patients with cow milk allergy (DMA) were enrolled into this study. The diagnosis of CMA was confirmed by positive milk challenge test, positive skin prick test and serum specific IgE (>3.5 kU/L) following published procedures [32]. Patients had severe organ diseases, or autoimmune diseases, or cancer, were excluded. The demographic data are presented in Table 1. In addition, 72 healthy volunteer subjects were enrolled into this study.

**Table 1:** Demographic data

Parameters	FA patients	Healthy subjects
Number	32	72
Males	15	36
Females	17	36
Age (years)	27.9 ± 12.6	25.8 ± 11.2
FA history (years)	3.6 ± 3.9	No
Eosinophils (%)	5.6 ± 3.8	1.8 ± 0.5*
sIgE (kU/L)	25.6 ± 18.8	ND
Serum IL-4 (pg/ml)	55.6 ± 18.4	12.8 ± 3.2*
Serum IL-5 (pg/ml)	64.8 ± 21.2	15.8 ± 3.5*
Serum IL-13 (pg/ml)	184.6 ± 32.5	25.8 ± 4.9*

FA: Food allergy. ND: Not detectable.

* , p<0.01, compared with FA patients. Eosinophils: Percentage of eosinophils in white blood cells. The data are presented as mean ± SD.

### Peripheral blood collection

From each subject, 40 ml peripheral blood was collected via ulnar vein puncture. Peripheral blood mononuclear cells (PBMC) were isolated from the blood samples by gradient density centrifugation. CD19^+^ B cells were purified from the PBMCs by magnetic cell sorting (MACS) with commercial reagent kits following the manufacturer's instructions. The purity of the isolated B cells was greater than 98% as checked by flow cytometry.

### Cell culture

The B cells were cultured with RPMI1640 medium supplemented with 10% fetal bovine serum, 100 U/ml penicillin, 0.1 mg/ml streptomycin, 2 mM L-glutamine and anti-CD40 (10 ng/ml; to prevent B cell apoptosis). The medium was changed in 2-3 days. The cell viability was greater than 99% before used for further experiments assessed by Trypan blue exclusion assay.

### Flow cytometry

In surface staining, cells were incubated with fluorochrome labeled antibodies as denoted in the figures or isotype IgG for 30 min at 4°C. After washing with phosphate-buffered saline (PBS), the cells were fixed with 2% paraformaldehyde for 1 h at room temperature. The cells were washed with PBS and followed by incubating with fluorochrome labeled antibodies or isotype IgG for 30 min at 4°C. After washing with PBS, the cells were analyzed with a flow cytometer (FACSCanto II; BD Biosciences). The data were analyzed with software flowjo with the data of isotype IgG staining as the gating reference.

### Real time quantitative RT-PCR (RT-qPCR)

The total RNA was extracted from cells with the TRIzol reagents and converted to cDNA with a reverse transcription kit. The cDNA was amplified in the CFX96 Touch™ Real-Time PCR Detection System (Bio-Rad) with the SYBR Green Master Mix. The results were calculated with the 2^−ΔΔCt^ method and presented as fold change against controls. The primers used in the present study include IL-10 (gttctttggggagccaacag and gctccctggtttctcttcct), HDAC11 (gtcttgcctgttcagtgcaa and tgcatccctgatttccacct) and β-actin (cgcaaagacctgtatgccaa and cacacagagtacttgcgctc). The primers were synthesized by Enke Biotech (Shenzhen, China).

### Western blotting

The total proteins were extracted from the cells, fractioned (50 μg/well) by SDS-PAGE (sodium dodecyl sulfate polyacrylamide gel electrophoresis), and transferred onto a PVDF membrane. After blocking with 5% skim milk for 30 min at room temperature, the membrane was stained with the primary antibodies as denoted in the figures or isotype IgG (used as controls) at 4°C overnight, and followed by staining with the second antibodies (conjugated with peroxidase) for 1 h at room temperature. The membrane was washed with TBST (Tris-buffered saline Tween 20) for 3 times after each time of incubation. The membrane was developed with ECL (enhanced chemiluminescence). The results were photographed with an imaging station (KODAK, ImagePro4000). The integrated density of the immune blots was assessed by ImageJ software.

### RNA interference (RNAi)

A portion of the cells was treated with shRNA kits (carried by lentiviral vector) to knock down the gene of A20 or HDAC11 following the manufacturer's instructions. The effects of RNAi were assessed by Western blotting.

### Luciferase reporter gene assay

The luciferase reporter genes of A20 were constructed by Genescript (Nanjing, China). The reporter genes were transfected into B cells following the manufacturer's instructions. In the reporter-gene analysis, the relevant light intensity was measured with a luminometer with a Luciferase kit. All assays were done in triplicate.

### Chromatin IP (ChIP)

ChIP was performed with the B cells and a reagent kit following the manufacturer's instructions. Briefly, cells were harvested from the culture and fixed with 1% formaldehyde for 15 min. The cells were then sonicated to shear DNA together with bound proteins into small fragments. Cell lysate was precleared by incubating with protein G-agarose beads for 2 h at 4°C. The samples were incubated at 4°C overnight with specific antibodies as denoted in the figures or isotype IgG, and precipitated by incubation with protein G agarose beads. The antibodies-DNA-protein complexes were collected by centrifugation. After cross-link-reversal and DNA purification, qPCR was performed with the samples and inputs. The primers of the promoter regions of IL-10 include ctgtgccaacgaagatcctc and aacattcgcctagagtcccc. The results are presented as relative changes against the input.

### Mice

Male C57/B6 mice (6-8 week old) were purchased from Beijing Experimental Animal Center (Beijing, China). The BC^A20−/−^ mice (mice with A20-deficient B cells) were purchased from the Huada Gene Biotech (Shenzhen, China). B cells were isolated from the BC^A20−/−^ mice (spleen and intestine) and analyzed by RT-qPCR and Western blotting. The results showed no A20 expression in the B cells (Figure 6). Mice were maintained in a pathogen-free environment and freely accessed to water and food.

### Development of FA mouse model

Following published procedures [33] with minor modification, C57BL/6 mice and BC^A20−/−^ mice were sensitized intraperitoneally with OVA (200 μg/mouse) mixing in 0.3 ml alum on day 0. The mice were boosted twice with the same treatment on days 3 and 9, respectively. Mice were challenged with OVA (500 μg/mouse) in 0.3 mL of saline intragastrically twice on days 10, 12 and 14, respectively. Mice then were killed 1 day after the last intragastric challenge (day 15).

### Assessment of allergic response in FA mouse model

#### Assessment of systemic response

The core temperature was recorded from each mouse by measuring the rectum temperature with a digital thermomitor 30 min after specific Ag challenge.

#### Recording diarrhea

Mice with diarrhea were counted during a period of 2-h after Ag challenge.

#### Measurement of serum IL-4 and OVA-specific IgE

Blood samples were collected from each mouse at the sacrifice. The sera were isolated via centrifugation. The serum levels of IL-4 and OVA-specific IgE were determined by enzyme-linked immunosorbent assay (ELISA) with commercial reagent kits following the manufacturer's instructions.

#### Counts mast cells and eosinophils in the intestinal mucosa

A segment of the jejunum was cut at the sacrifice and fixed with 4% formalin overnight. Paraffin sections were prepared and stained with 0.5% toluidine blue (for mast cell counts) or hymatoxylin & eosin (for eosinophil counts). Mast cells and eosinophils were counted on the sections under a light microscope in 20 randomly selected fields at ×400 magnification. The slides were coded. The observers were not aware of the code to avoid the observer bias.

#### Detection of Ag-specific CD4^+^ T cells

Small intestinal segments were excised at the sacrifice. The tissue was cut into small pieces (2×2×2 mm) and incubated with 1 mg/ml collagen IV for 2 h at 37°C. The lamina propria mononuclear cells (LPMC) were filtered through a cell strainer (40 μm) and harvested by centrifugation. CD4^+^ CD25^−^ T cells and dendritic cells (DC) were isolated from the cells with magnetic cell sorting kits following the manufacturer's instructions (cell purity = 99% as checked by flow cytometry). The T cells (labeled with CFSE) and DC were cultured in RPMI1640 medium in the presence of OVA (the specific Ag; 5 μg/ml) or bovine serum albumin (BSA, an irrelevant Ag, 5 μg/ml) for 3 days. The cells were collected from the culture and analyzed by flow cytometry, the CFSE-dilution assay. The proliferating cells were regarded as OVA-specific CD4^+^ T cells.

### Reconstitution of A20-sufficient B cells in BCA20^−/−^ mice

B cells were isolated from C57/B6 mouse spleen by magnetic cell sorting with a commercial reagent kit. The purity of isolated B cells was greater than 99% as checked by flow cytometry. The BC^A20−/−^ mice were received 10^6^ B cells/mouse on day 0, and day 7 respectively via tail vein injection. After the sacrifice, IL-10^+^ B cells were detected in the spleen and intestine of the recipient mice (Figure 6).

### Statistics

All the data are normally distributed as tested by Norm.Dist in MS Excel. The data are presented as mean ± SD. Student t test was used to determine the difference between two groups. If more than two groups, two-way analysis of variance (ANOVA) along with Bonferroni correction was used. p<0.05 was set as a significant criterion.
